# Prognostic Performance of a Modified TRI-SCORE Incorporating RV–PA Uncoupling After Transcatheter Tricuspid Valve Interventions

**DOI:** 10.3390/jcdd13060250

**Published:** 2026-06-05

**Authors:** Mhd Nawar Alachkar, Johannes Schlegl, Marwin Bannehr, Tanja Kücken, Michael Lichtenauer, Vera Paar, Michael Neuß, Anja Haase-Fielitz, Christoph Edlinger, Christian Butter

**Affiliations:** 1 Department of Cardiology, University Hospital Heart Center Brandenburg, 16321 Bernau bei Berlin, Germany; johannes.schlegl@immanuelalbertinen.de (J.S.); marwin.bannehr@immanuelalbertinen.de (M.B.); tanja.kuecken@immanuelalbertinen.de (T.K.); michael.neuss@immanuelalbertinen.de (M.N.); 2 Faculty of Medicine, Brandenburg Medical School (MHB) Theodor Fontane, 16816 Neuruppin, Germany; 3 Clinic of Internal Medicine II, Department of Cardiology, Paracelsus Medical University of Salzburg, 5020 Salzburg, Austria; m.lichtenauer@salk.at (M.L.); l v.paar@salk.at (V.P.); 4 Institute of Social Medicine and Health System Research, Otto von Guericke University Magdeburg, 39120 Magdeburg, Germany

**Keywords:** tricuspid valve intervention, TRI-SCORE, RV-PA coupling

## Abstract

Background: The TRI-SCORE was developed to predict mortality after tricuspid valve surgery and has demonstrated prognostic value in patients undergoing transcatheter tricuspid valve interventions (TTVI). Right ventricular–pulmonary arterial (RV–PA) uncoupling assessed by the TAPSE/sPAP ratio has emerged as a prognostic marker in selected populations; however, its incremental value within established risk scores remains unclear. Methods: In this prospective single-centre cohort, 109 patients undergoing TTVI were included. The original TRI-SCORE was calculated for all patients. A modified TRI-SCORE was proposed by substituting the definition of right ventricular dysfunction based on TAPSE with RV–PA uncoupling, defined as TAPSE/sPAP <0.406 using invasively measured systolic pulmonary artery pressure. The endpoints were 12-month all-cause mortality and a combined endpoint of death or cardiovascular rehospitalization. Results: At 12 months, all-cause mortality occurred in 19.3% of patients, and the combined endpoint in 40.4%. Both original and modified TRI-SCOREs were significantly associated with 12-month mortality (OR 1.80 per point increase, 95% CI 1.30–2.48; *p* < 0.001 and OR 1.81 per point increase, 95% CI 1.31–2.49; *p* < 0.001, respectively) and demonstrated comparable discrimination (AUC 0.78 for both; DeLong *p* = 0.90). Furthermore, both scores were significantly associated with the combined endpoint (TRI-SCORE: OR 1.36 per point increase, 95% CI 1.08–1.71; *p* = 0.008; modified TRI-SCORE; OR of 1.33 per one-point increase, 95% CI 1.07–1.66; *p* = 0.009) with modest and comparable AUCs (~0.65). Conclusion: In patients undergoing TTVI, replacing TAPSE with RV–PA uncoupling did not improve the prognostic performance of the TRI-SCORE for mortality or combined clinical outcomes.

## 1. Introduction

The TRI-SCORE was initially developed to predict mortality after isolated tricuspid valve surgery [1]. Alongside other dedicated risk scores such as the TRI-VALVE score [2], the TRI-SCORE has also demonstrated prognostic value in patients undergoing transcatheter tricuspid valve interventions (TTVI) [3,4]. However, Adamo et al. suggested that the prognostic performance of the TRI-SCORE in TTVI may be suboptimal, highlighting the need for further refinement of this score in this population [5]. The TRI-SCORE is composed of multiple clinical and echocardiographic parameters, including right ventricular dysfunction defined by tricuspid annular plane systolic excursion (TAPSE) < 17 mm [1].

However, RV-PA uncoupling, defined as a TAPSE/sPAP ratio < 0.406, has been shown to be an independent predictor of all-cause mortality in patients undergoing TTVI [6]. A modified TRI-SCORE was proposed, in which right ventricular dysfunction was defined by RV–PA uncoupling instead of TAPSE alone. Despite its physiological relevance, it remains unclear whether RV–PA uncoupling provides incremental prognostic information beyond established composite risk scores such as the TRI-SCORE. Therefore, integrating RV–PA coupling into existing risk stratification models may represent a promising approach to refine prognostic assessment in patients undergoing TTVI. This study aimed to compare the performance of the original and modified TRI-SCOREs in patients undergoing TTVI.

## 2. Methods

This single-centre prospective observational study enrolled patients undergoing TTVI. The study was initiated in November 2022. Between November 2022 and December 2024, a total of 112 patients were included. All patients had symptomatic severe TR as defined by current guidelines [7,8] and were discussed in the heart team. All patients underwent comprehensive transthoracic echocardiography (TTE). LV-EF was calculated using Simpson’s biplane method. TAPSE was assessed using M-mode in the apical four- chamber view. TR severity was assessed using an integrative multiparametric approach as recommended [9]. Furthermore, patients underwent invasive right heart catheterisation (RHC) as part of the standard preprocedural assessment. In all cases, heart team recommended proceeding with TTVI. According to heart team recommendations, patients underwent T-TEER, heterotopic, or orthotopic tricuspid valve replacement. All procedures were performed under general anaesthesia using fluoroscopic and echocardiographic guidance. The TRI-SCORE was calculated according to the original published definition [1] using the dedicated online calculator (https://www.tri-score.com, accessed on 2 January 2026). In addition, a modified TRI-SCORE was proposed, in which right ventricular dysfunction was defined by right ventricular–pulmonary arterial (RV–PA) uncoupling, assessed as the ratio of tricuspid annular plane systolic excursion to invasively measured systolic pulmonary artery pressure (TAPSE/sPAP), instead of TAPSE alone. RV–PA uncoupling was defined using an invasive TAPSE/sPAP cut-off of 0.406, as previously described by Berner et al. [4]. Due to missing data on invasive sPAP measurements, three patients were excluded, resulting in a final study population of 109 patients. All patients provided written informed consent prior to inclusion, and the study was approved by the local ethics committee (approval number E-01-20190503). The endpoints were 12-month all-cause mortality and a combined endpoint of death or cardiovascular rehospitalization. Follow-up data were obtained through clinical visits and/or telephone interviews at 12 months. Rehospitalization was defined as any unplanned cardiovascular hospital admission during follow-up.

### Statistical Analysis

The association of the original and the modified TRI-SCOREs with 12-month all-cause mortality was assessed using univariable logistic regression analysis, with results reported as odds ratios (ORs) per one-point increase with 95% confidence intervals (CIs). Discriminatory performance for 12-month mortality was evaluated using receiver operating characteristic (ROC) curve analysis and quantified by the area under the curve (AUC). The AUCs of the original and modified TRI-SCOREs were compared using the DeLong test for paired ROC curves. A two-sided *p*-value < 0.05 was considered statistically significant. Consistent with prior analyses [4], the same analyses were performed for the entire cohort and for patients with intermediate or high-risk TRI-SCORE (i.e., TRI-SCORE ≥ 4). Statistical analyses were performed using R version 4.2.3 (R Foundation for Statistical Computing, Vienna, Austria).

## 3. Results

TTVI included TEER in 88 patients, TricValve in 17 patients, and EVOQUE in 4 patients. The modified TRI-SCORE differed from the original TRI-SCORE in 19 of 109 patients (17%), resulting in upward reclassification (one point increase) in 10 patients and downward reclassification (one point decrease) in 9 patients. Baseline characteristics of the study population are summarized in Table 1.

### 3.1. Mortality

**Figure 1:**
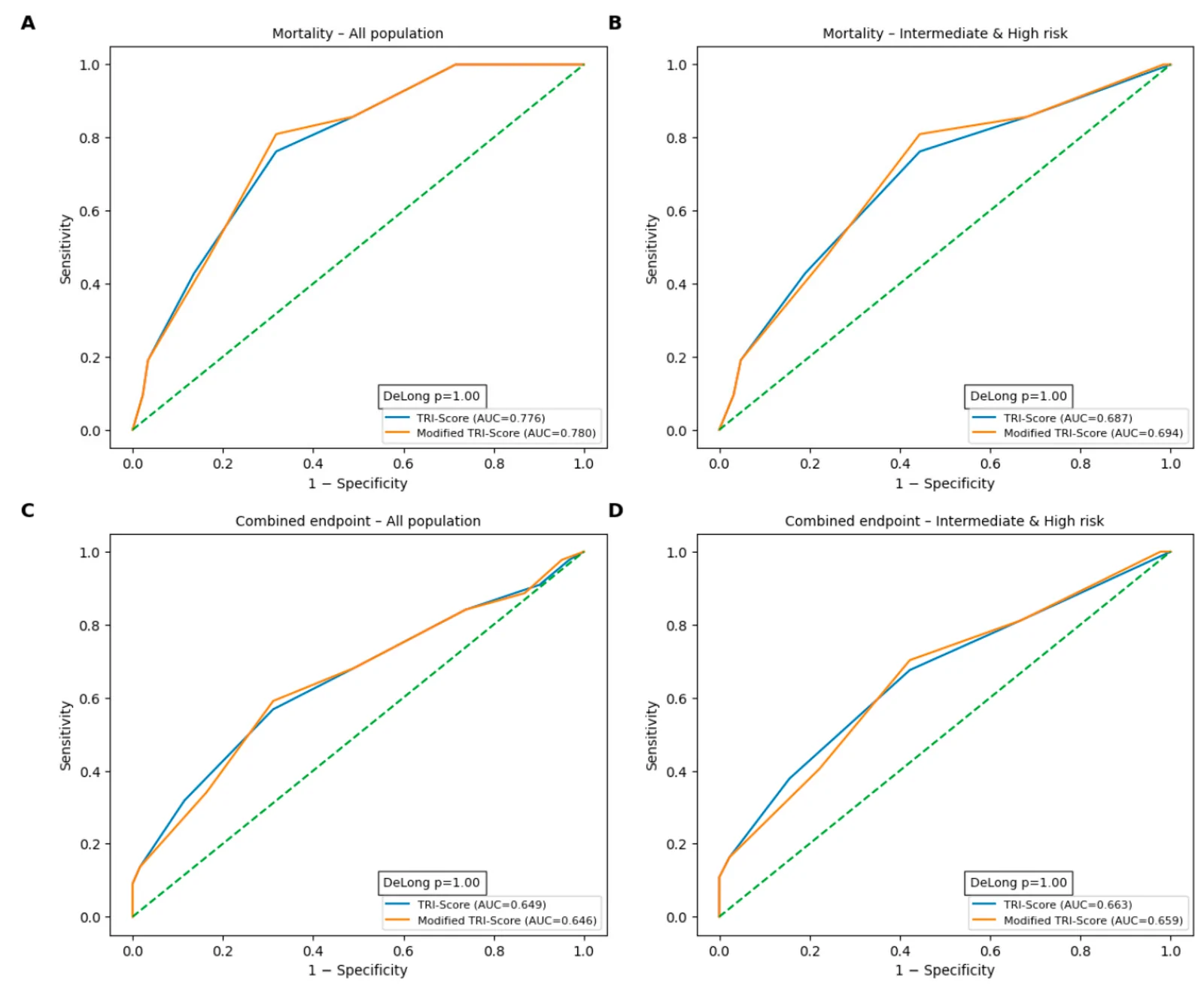
(ROC) curves comparing the original and modified TRI-Score for prediction of clinical outcomes. Upper row: 12-month mortality in the overall study population (**A**) and in patients with intermediate and high risk (**B**). Lower row: ROC analyses for the combined endpoint of death or rehospitalization in the overall population (**C**) and in patients with intermediate and high risk (**D**).

At 12 months, all-cause mortality occurred in 21 of 109 patients (19.3%). After exclusion of low-risk patients according to the original TRI-SCORE definition, mortality increased to 25.3% (21/83). In univariable logistic regression analysis, both original and modified TRI-SCOREs were significantly associated with 12-month mortality (OR 1.80 per point increase, 95% CI 1.30–2.48; *p* < 0.001 and OR 1.81 per point increase, 95% CI 1.31–2.49; *p* < 0.001, respectively). In ROC analysis, both scores showed good discriminatory performance for 12-month mortality, with an AUC of 0.77 (95% CI 0.651–0.900; *p* < 0.001) for the original and 0.78 (95% CI 0.657–0.904; *p* < 0.00) for the modified score, with no significant difference between the AUCs (DeLong test- *p* = 0.9). ROC curves are demonstrated in Figure 1.

After exclusion of low-risk patients, two patients were reclassified from intermediate to high risk, whereas one patient was reclassified from high to intermediate risk. Both scores remained significantly associated with 12-month mortality (OR 1.57 per point increase, 95% CI 1.09–2.24; *p* = 0.024 and OR 1.59 per point increase, 95% CI 1.11–2.27; *p* = 0.012), and both demonstrated good discriminatory performance in ROC analysis, with an AUC 0.68 (95% CI 0.548–0.826; *p* = 0.009) for the original and 0.69 (95% CI 0.556–0.833; *p* = 0.006) for the modified score respectively without significant difference between both AUCs (DeLong test- *p* = 1.00) (Figure 1).

### 3.2. Combined Endpoint (Death or Cardiovascular Rehospitalization)

At 12 months, the combined endpoint of death or rehospitalization was observed in 44 of 109 patients (40.4%). After exclusion of low-risk patients, the combined endpoint increased to 43.3% (36/83).

In univariable logistic regression analysis, both the original TRI-SCORE and the modified TRI-SCORE were significantly associated with the combined endpoint (OR 1.36 per point increase, 95% CI 1.08–1.71; *p* = 0.008 and OR of 1.33 per one-point increase, 95% CI 1.07–1.66; *p* = 0.009). ROC analysis demonstrated comparable discriminatory performance of both the original and modified scores, with a modest AUCs of 0.65 (95% CI 0.541–0.757; *p* = 0.007 and 95% CI 0.538–0.754;, *p* = 0.008, respectively), with no significant difference in the DeLong test (DeLong *p* = 1.00). After exclusion of low-risk patients, both the original and the modified TRI-SCORE were still significantly associated with the combined endpoint (OR 1.41, 95% CI 1.10–1.80; *p* = 0.006 and OR 1.39, 95% CI 1.09–1.77; *p* = 0.008). Discriminatory performance was comparable between both original and modified scores, with identical AUCs of 0.66 (95% CI 0.544–0.782), *p* = 0.007 and 95% CI 0.540–0.779; *p* = 0.009) with no significant difference between the two scores in the DeLong test (*p* = 1.00) (Figure 1).

## 4. Discussion

The present study demonstrates that the TRI-SCORE retains its prognostic performance in patients undergoing TTVI. TAPSE remains one of the most widely used and guideline-recommended echocardiographic parameters for the assessment of RV systolic function due to its simplicity and reproducibility. However, TAPSE alone may not fully reflect RV adaptation to increased afterload in patients with pulmonary hypertension or severe TR. Therefore, RV–PA coupling assessed by the TAPSE/sPAP ratio has emerged as a more integrative functional parameter and has demonstrated prognostic value in patients undergoing TTVI and in other heart failure populations [6,10,11]. However, substituting the original definition of right ventricular dysfunction with RV–PA uncoupling assessed by TAPSE/sPAP did not improve the prognostic performance of the score, which may be explained by several factors. First, the TRI-SCORE is a multivariable score incorporating several clinical and echocardiographic parameters, and the addition or substitution of a single parameter may not necessarily translate into incremental prognostic information. In addition, methodological differences in the assessment of RV–PA uncoupling may contribute to the lack of incremental value observed in our study. While previous studies [6] assessing TAPSE/sPAP predominantly relied on echocardiographically derived systolic pulmonary artery pressure, which may be underestimated in patients with severe TR, pulmonary pressures in our cohort were measured invasively, which may limit the applicability of previously proposed cut-offs. This difference in measurement approach may partly explain the lack of additional prognostic value observed in the modified score.

According to prior analyses [4] suggesting a better performance of the score in patients with intermediate and high risk, additional analyses were performed in this subgroup. Notably, the modified score differed from the original score in only three patients and did not demonstrate superior performance compared with the original score.

Furthermore, beyond classical risk scores, additional parameters integrating TR severity and right ventricular remodelling are increasingly being investigated for outcome prediction in patients undergoing TTVI. In this context, the concept of proportional and disproportionate TR based on the EROA/RVEDD ratio has recently demonstrated prognostic relevance after transcatheter tricuspid valve interventions [12].

Taken together, these findings suggest that while RV–PA uncoupling represents an important pathophysiological concept and a prognostic marker in many populations [6,10,11], its incorporation into the TRI-SCORE does not improve risk stratification in patients undergoing TTVI. The original TRI-SCORE, therefore, remains a practical and reliable tool for risk assessment in this setting.

### Limitations

This study is limited by its single-centre design and moderate sample size. In addition, the study population was heterogeneous and included various TTVI modalities, which may differ regarding clinical outcomes and prognostic implications. Furthermore, the proposed modification of the TRI-SCORE should be considered exploratory and hypothesis-generating. Larger multicentre studies are warranted to further evaluate potential refinements of risk stratification strategies in patients undergoing TTVI.

## 5. Conclusions

The TRI-SCORE predicts mortality and a combined endpoint of death or cardiovascular hospitalization in patients undergoing transcatheter tricuspid valve interventions. A proposed modified score incorporating the use of RV-PA uncoupling instead of conventional right ventricular dysfunction did not improve the performance of the original score.

## Figures and Tables

**Table 1:** Baseline characteristics and outcome.

Baseline Characteristics	
Age (years)	80.7 ± 6.6
Male, n (%)	52 (47.7)
DM, n (%)	39 (35.7)
CAD, n (%)	54 (49.6)
Arterial hypertension, n (%)	83 (76.1)
eGFR (ml/min)	41.7 ± 14.2
eGFR < 30 ml/min, n (%)	23 (21.1)
Elevated Bilirubin	24 (22.0)
Atrial fibrillation, n (%)	105 (96.3)
CIED, n (%)	37 (33.9)
LV-EF, %	55 (45–60)
TAPSE, mm	16 (14–19)
sPAP, mmHg	42 (33.5–50)
TAPSE/sPAP	0.38 (0.30–0.49)
TR severity †	4 (3–5)
Type of intervention, n (%)	
TEER	88 (80.8)
TricValve	17 (15.6)
Evoque	4 (3.6)
Outcome at one year	
Mortality at one-year follow up, n (%)	21 (19.3)
combined endpoint at one-year follow-up, n (%)	44 (40.4)
TR severity at one-year follow-up †	1.5 (1.5–2.5)

Abbreviations: DM: Diabetes milletus, CAD: coronary artery disease, eGFR: estimated glomerular filtration rate, CIED: Cardiac Implantable Electronic Devices, LV-EF: Left Ventricular Ejection Fraction, TAPSE: Tricuspid Annular Plane Systolic Excursion, sPAP: systolic pulmonary artery presssure, TR: Tricusoid Regurgitation. † TR severity was graded according to the 5-grade classification scheme for TR severity, and values were presented numerically as follows: 1 = mild, 2 = moderate, 3 = severe, 4 = massive, and 5 = torrential.

## Data Availability

The data presented in this study are available on request from the corresponding author.
